# Optimization of tribo-mechanical properties of kenaf/jute-SiC hybrid composites using integrated grey-fuzzy approach

**DOI:** 10.1038/s41598-025-11340-z

**Published:** 2025-07-19

**Authors:** Aravindh Murugan, Debabrata Barik, Rasan Sarbast Faisal, Makeshkumar Mani, Milon Selvam Dennison, Seepana Praveenkumar, Saravanan Rajendran

**Affiliations:** 1 Department of Mechanical Engineering, Karpagam Academy of Higher Education, Coimbatore, 641021 India; 2 Centre for Energy and Environment, Karpagam Academy of Higher Education, Coimbatore, 641021 India; 3 Department of Petroleum Engineering, College of Engineering, Knowledge University, Erbil, 44001 Iraq; 4 Department of Petroleum Engineering, Al-Kitab University, Altun Kupri, 36001 Iraq; 5 Department of Mechanical Engineering, KPR Institute of Engineering and Technology, Coimbatore, 641407 India; 6 Department of Mechanical Engineering, Kampala International University, Western Campus, Ishaka-Bushenyi, Uganda; 7 Department of Nuclear and Renewable Energy, Ural Federal University, Ekaterinburg, 620002 Russia; 8 Instituto de Alta Investigación, Universidad de Tarapacá, Arica, 1000000 Chile

**Keywords:** Tribological characteristics, Mechanical properties, AI with GRG, AI with GFG, Kenaf/jute-SiC, Hybrid composites

## Abstract

An increasing need for wear-resistant hybrid materials has prompted researchers to develop alternative materials that comprise different reinforcements and fillers. In recent studies, the combination of natural fibers and ceramic fillers has resulted in hybrid composites with improved tribological characteristics for automotive and aircraft applications. Even though natural fibers have some disadvantages, the combined effect of natural fibers and ceramics with suitable multi-response optimization techniques can overcome the limitations and provide a composite with enhanced mechanical and tribological properties at minimal cost. This paper explores the wear behavior of silicon carbide (SiC) filled kenaf/jute-based hybrid composite. In addition, a multi-response optimization technique combining the fuzzy model interference system and grey relation analysis (GRA) is adopted in the current work. Design of experiments was carried out with the Taguchi L27 orthogonal array (OA) to yield the minimum wear rate and coefficient of friction (COF). This study demonstrated that the highest gray relational grade (GRG) and gray fuzzy grade (GFG) value of 0.804 and 0.801, respectively to obtain the optimal operating parameters of 1 wt% SiC, 30 wt% reinforcement, 1500 m sliding distance, 1.5 m/s sliding speed, and 15 N load. Scanning electron microscope (SEM) revealed worn-out surface mechanisms, fine debris, and the ploughing effect, which play a significant role in begetting the desired wear characteristics. Furthermore, the best combination of particulate hybrid composite was tested for mechanical characteristics in dry and wet conditions.

## Introduction

In recent times, the automotive and aerospace industries have actively sought innovative tribo-based materials to replace traditional synthetic materials for extreme service and harsh environments^1^. Natural fibers are a possible alternative for substituting materials in different engineering applications owing to being environmentally safe, biodegradable, cheap, having high specific strength, a high strength-to-weight ratio, and productivity, corrosion resistance, noise reduction, and self-lubrication^2^. Many components are subject to friction and wear phenomena, including brakes, shafts, cams, impellers, conveyors, transmission belts, bushings, bearings, impellers, cylinder liners, and drum brakes. The mechanism of the worn-out surface is composed of complex tribo failure phenomena, which constitute a number of parameters such as interfacial surface reactions, matrix properties, fibers, fillers, and harsh environments^3^. Brailson Mansingh et al.^4^ studied the influence of SiC nano filler on the mechanical and tribological properties of 5 wt% alkaline-treated areca fruit husk hybrid composite. They found that by varying SiC filler nano particles from 1 to 3 wt%, wear resistance and mechanical and water absorption increased significantly. However, beyond 3 wt%, the trend declined due to particle agglomeration, which promotes weakened interface, porosity, and subsequent swelling, ultimately leading to diminished overall performance. Similarly, Hariharan et al. and Saravanakumar et al.^5,6^ reported that agglomeration and heterogeneous distribution of SiC nanoparticles in 5 wt% alkaline-treated Areca/Tamarind and Agave Tequilana biowaste with 3 wt% SiC nanoparticles reduced their mechanical and tribological properties. Likewise, Thooyavan et al.^7^ investigated the effect of micro- and nano SiC particles on the mechanical, wear, and moisture absorption of basalt bidirectional mat/vinyl ester composites. It is noticed that the advantage of wear, mechanical, and moisture absorption of the ceramic filled hybrid composite decreases when more than 3 wt% SiC filler is added due to poor dispersion and agglomeration of the filler particles.

In recent years, numerous researchers have discovered that the addition of various inorganic nanoparticles, such as Al_2_O_3_, SiC, TiC, ZnO, MgO, as well as graphite, carbon nanotubes, and nanofibers, along with agro wastes like fly ash, red mud, rice husk, bagasse, and date fruit seed, to epoxy matrices leads to improved load-bearing capacity, reduced friction coefficient, and enhanced wear and moisture resistance^8–18^. Among various fillers, SiC exhibits superior wear and abrasion resistance, excellent chemical inertness, and an exceptionally high melting point. Furthermore, tribo film leads to the introduction of a new perspective on the frictional and wear behavior in the tribo phenomenon environment, which plays a critical and predominant role in understanding frictional behavior^19^. Therefore, the understanding of tribological characteristics in ceramic filled hybrid composites has been considered a key role in different service environments, such as loads, sliding speed, and sliding distance in view of different fillers and reinforcements for a sustainable environment.

In this context, Chin & Yousif et al.^20^ explored the wear performances of kenaf/epoxy composites with various orientations of fibers, antiparallel, parallel, and normal for mild load bearing application. The research concluded that the use of kenaf fibers in random orientation effectively increased frictional resistance by around 85% due to the well-distributed fibers in the epoxy matrix. Likewise, Kumar et al.^21^ tested the influence of fly ash filler on the tribological and abrasion wear attributes of alkali-treated Himalayan nettle fiber composites. Parameters were changed as follows: fiber loading (5–15%), normal load (10–30 N), content of fly ash (5–15%), and speed of sliding (50–150 rpm). ANOVA was utilized to determine the degree of each parameter for improving tribological performance, specifically COF.

According to their results, applied load and sliding distance were found to be the most critical parameters influencing tribological performance, both contributing significantly to wear and friction resistance. Sumesh et al.^22^ investigated the tribological behavior of TiO₂-reinforced pineapple/sisal hybrid composites with a Taguchi L_27_ orthogonal array. The research indicated that both pineapple and sisal fibers contributed significantly to reducing wear and friction, highlighting their synergistic influence on tribological behaviour. Kannan et al.^23^ conducted wear study on banana fiber/fly ash-reinforced polyester composite through control factors including filler percentage (1, 3, and 5 vol.%), sliding speed (300, 500, and 700 rpm), applied load (20, 40, and 60 N), and time of sliding (5, 10, and 15 min and reported that applied load significantly contributed towards the maximum impact on wear rate as evident through a maximum value of F-statistic 17.70.

Similarly, Narish et al.^24^ investigated the tribological properties of kenaf fiber-reinforced composites. After analyzing results, it was observed that the incorporation of kenaf fiber produced a 78% reduction in the specific wear rate, showcasing its ability as a natural reinforcement for wear-resistant composite materials.

Experimental investigations have consistently demonstrated that external harsh conditions such as high applied loads, elevated sliding speeds, and extended sliding distances have a pronounced impact on the wear behavior of particulate-reinforced hybrid composites, particularly when analyzed through computational methods. These findings highlight the necessity for deeper exploration into the interactions between frictional behavior and wear mechanisms under extreme service environments. Therefore, it becomes essential to realize the frictional behavior and wear mechanisms of composites under extreme conditions, such as high loads, high sliding velocities, high operating temperatures, and water-lubricated interfaces, by using sophisticated computational and simulation methods^25^.

Various researchers have adopted several methodologies to analyze and optimize tribological behavior in composite materials. Notable among these are the Taguchi method^26,27^, Response Surface Methodology (RSM)^28^ and Gray Relational Analysis (GRA)^29,30^. Additionally, soft computing techniques such as Artificial Neural Networks (ANN) and Adaptive Neuro-Fuzzy Inference Systems (ANFIS), along with artificial intelligence (AI)-driven metaheuristic algorithms-such as Particle Swarm Optimization (PSO), Genetic Algorithms (GA), and Teaching–Learning-Based Optimization (TLBO)^31,32^, have been employed to investigate the influence of various control parameters on wear and frictional performance.

But, the gray-based fuzzy logic he gray-based fuzzy logic method has proven to be an exceptionally good tool. This method captures the capability to learn from uncertain, imprecise, or vague training data-similar to ANN, yet uses the structure of a Fuzzy Inference System (FIS) to model and forecast the wear behavior of particulate hybrid fiber-reinforced composites with great accuracy^33,34^. For instance, Sinha et al.^35^ examined the wear behavior of areca fiber/red mud-filled polymer composites using a hybrid modeling technique based on Response Surface Methodology (RSM) along with fuzzy logic. The objective was to determine the optimal condition under which the wear rate could be minimized. The results showed that increasing the red mud filler content from 4 to 8% by weight had a pronounced effect on improving the wear resistance of the composite as a result of better dispersion and load-carrying ability of the filler. Similarly, Bhowmik et al.^36^ introduced an artificial intelligence (AI)-guided fuzzy logic method for optimizing the three most important tribological output responses: wear rate, friction force, and coefficient of friction (COF). Gray fuzzy logic was used to execute the optimization process. ANOVA was employed to verify model reliability, which found the applied load as the most significant parameter to obtain an optimal Gray Fuzzy Relation (GFR). Likewise, Srinivasan & Raajarajan et al.^37^ compared the wear behavior of coconut coir-reinforced composites with a fuzzy inference system with triangular membership functions for the control factors. Their findings showed that the gray-fuzzy hybrid optimization method provided very accurate predictions and facilitated efficient identification of optimal combinations of input parameters for improved tribological performance. Similarly, Jagadeesh et al.^38^ constructed the fuzzy model to predict the tribological behavior of polymer composite and found that the developed model predicts wear results with a 3.12% error and friction coefficient with a 2.19% error.

From the in-depth literature survey presented above, it is clear that the identification and optimization of the appropriate process variables are vital in dealing with the multi-response tribological behavior of composite materials. Optimizing a performance parameter often affects or undermines others, thus requiring balanced and systematic attention. In this regard, the current research applies an integrated fuzzy logic–based Gray Relational Analysis (GRA) approach to handle the embedded uncertainty and variability in experimental results, and hence determine the best and safest operating conditions. The originality of this work resides in the construction of a highly integrated and strong multi-response optimization model. In addition to providing accurate prediction and optimization under situations of complex, nonlinear interactions, the model guarantees improved operational reliability and safety under extreme environments. Additionally, the suggested methodology effectively minimizes experimental work and corresponding costs, yet with minimal discrepancy between predicted and experimental values, highlighting its practical applicability and industrial utility.

## Materials and methodology

### Materials used

**Fig. 1:**
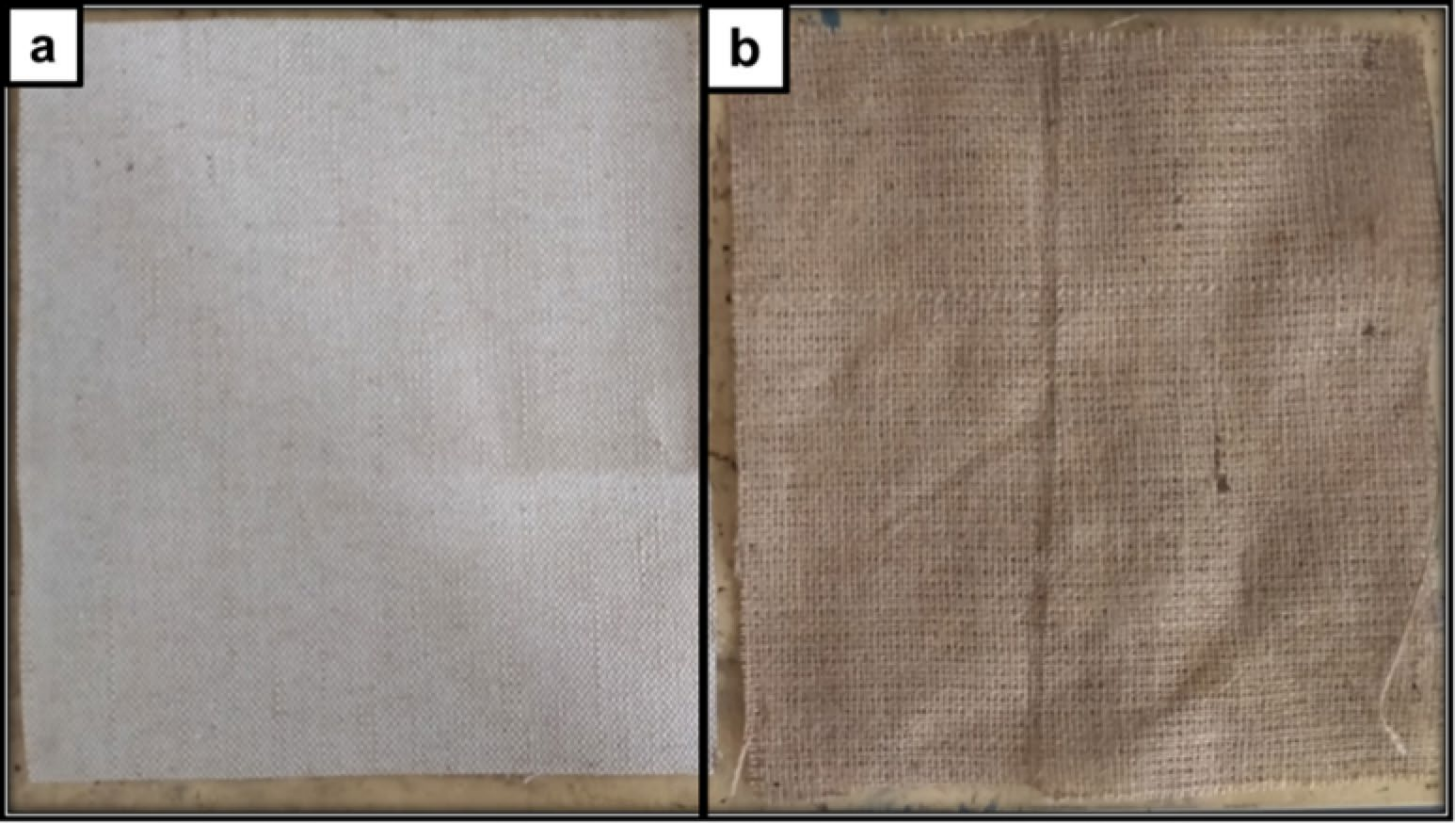
(**a**) Kenaf woven mat (**b**) Jute woven mat.

Figure 1a,b show the Kenaf woven mat and the Jute woven mat used as reinforcement material. As matrix material, epoxy Araldite LY556 grade material was utilized. The epoxy was blended with anhydride hardener at a 10:1 ratio (HY951 grade). The hardeners were chosen for their excellent adhesive characteristics. Dimethylformamide (DMF) and acetone were purchased from Loba Chemie, India, for the homogeneous dispersion of SiC. The reinforcement materials were utilized as jute and kenaf fiber, which are known for their excellent mechanical strength, high thermal stability, and long-lasting properties. SiC (15 µm) powder was used as the filler material purchased from Sigma-Aldrich U.S. and well-known for their high melting point, chemical stability, and resistance to wear and tear. The properties of reinforcements and matrix and fillers are listed in Table 1.

**Table 1:** Physical properties of fiber and resin.

Raw materials	Characteristics	
Epoxy resin	Grade:	LY556
	Chemical name:	Bisphenol-A-diglycidyl-ether
	Appearance:	Pale yellow liquid is clear,
	Viscosity:	10,000–12,000 (MPa.s) (25˚C)
	Density:	1.1–1.2 g/cm^3^
Hardener	Grade:	HY 951
	Chemical name:	2-amineethylethane-1, 2-diamin
	Appearance:	Visual, clear
	Specific gravity:	0.98 g/cm^3^ (at 20˚C)
Kenaf fiber	Type:	Non-woven mat
	Density:	1300 g/cm^3^
Jute fiber	Type:	Non-woven mat
	Density:	1.46 g/cm^3^
SiC	Type:	Powder
	Density:	0.1 g/cm^3^
	Surface area :	200 m^2^/g

### Experimental design (DoE)

The Taguchi design of experiments methodology was employed to assess the impact of independent parameters on dependent parameters. The current study is organized into five factors, including the weight of fillers, the weight of reinforcement, normal load, sliding distance, and sliding speed, each with three distinct levels, as presented in Table 2. An L_27_ orthogonal array was generated utilizing MINITAB 18 software, referring to the data given in Table 3. The experimentation, based on ASTM G99, was conducted on a Pin-on-disk apparatus with a specimen size of 30 mm × 10 mm, and repeated three times.

**Table 2:** Factors and their level.

Levels	Factors				
	Gr/SiC wt%	Reinforcement (Kenaf/Jute) wt%	Sliding distance (m)	Sliding speed (m/s)	Load
Notations	A	B	C	D	E
1	1	20	500	0.5	5
2	2	30	1000	1	10
3	3	40	1500	1.5	15

**Table 3:** L27 orthogonal array.

Exp. No	Parameter Inputs Factors				
	Gr/SiC wt%	Reinforcement (Kenaf/Jute) wt%	Sliding distance (m)	Sliding speed (m/s)	Load
1	1	2	500	0.5	5
2	1	2	1000	1	10
3	1	2	1500	1.5	15
4	1	3	500	1	15
5	1	3	1000	1.5	5
6	1	3	1500	0.5	10
7	1	4	500	1.5	10
8	1	4	1000	0.5	15
9	1	4	1500	1	5
10	2	2	500	0.5	5
11	2	2	1000	1	10
12	2	2	1500	1.5	15
13	2	3	500	1	15
14	2	3	1000	1.5	5
15	2	3	1500	0.5	10
16	2	4	500	1.5	10
17	2	4	1000	0.5	15
18	2	4	1500	1	5
19	3	2	500	0.5	5
20	3	2	1000	1	10
21	3	2	1500	1.5	15
22	3	3	500	1	15
23	3	3	1000	1.5	5
24	3	3	1500	0.5	10
25	3	4	500	1.5	10
26	3	4	1000	0.5	15
27	3	4	1500	1	5

Previous studies reported that with the enhancement of nano filler content, there might be a high occurrence of particle agglomeration in the polymer matrix, leading to a suspension in the case of additional nano filler presence. SiC fillers were used at (1, 3, and 5%) by weight. Because the filler content was 1–3%, lubrication reduced friction by 36% and wear by 32% owing to its C–C bond and strong Van der Waals forces. Furthermore, mechanical and tribological properties of the hybrid composite deteriorated when the SiC filler content exceeded 3 wt% due to the aggregation and agglomeration of the dispersion of filler particles^4,5,7^. In addition, kenaf/jute fibers (20, 30, 40%) by weight were used as reinforcement. Because of the unregulated factors increase in fiber clustering, porosity, fracture propagation, and micro voids in over 40% reinforcement. Adding 30% fiber reduced wear by 95% and friction by 39%^39,40^.

Sliding distance levels (500 m, 1000 m, and 1500 m) were chosen because the sliding distance beyond 1500 m, the frictional performance of the composite decreased due to continuous disk rotation and hard-to-hard contact with the counter surface causing hindering the thermal distribution of thermal stress across the interface. As a result, there was an increase in plastic deformation and delamination, which led to an increase in intensive acute wear loss and coefficient of friction and additional wear debris across the worn-out surface^41–43^.

Previous authors determined the effect of sliding speeds (0.5, 1, 1.5 m/s) on wear rate. Higher sliding speed might be associated with a high acute contact area resulting in superior contact temperature that can cause the soft matrix to melt at the counter interface, leading to a reduction in the matrix shear strength and more fiber pullout, and furrows on the fiber Surface. Therefore, fibers exposed to steel counter surfaces have revealed a formation of smoother dispersion of uniform tribofilm and greater adherence to the counterpart, which subsequently forms adequate wear debris and reduces the wear and coefficient of friction^44^.

Kishore et al.^45^ assessed the effects of load levels (5N, 10N, 15N) on wear tests. In similar to them, it can be understood that higher loads can cause increased wear and surface temperature as a result of the contact interface being altered from viscoelastic to plastic, thereby wear rate and coefficient of friction can increased by about 197.7% and 93.9%, respectively in comparison to the low load conditions (10N).

### Preparation of composite laminate

**Fig. 2:**
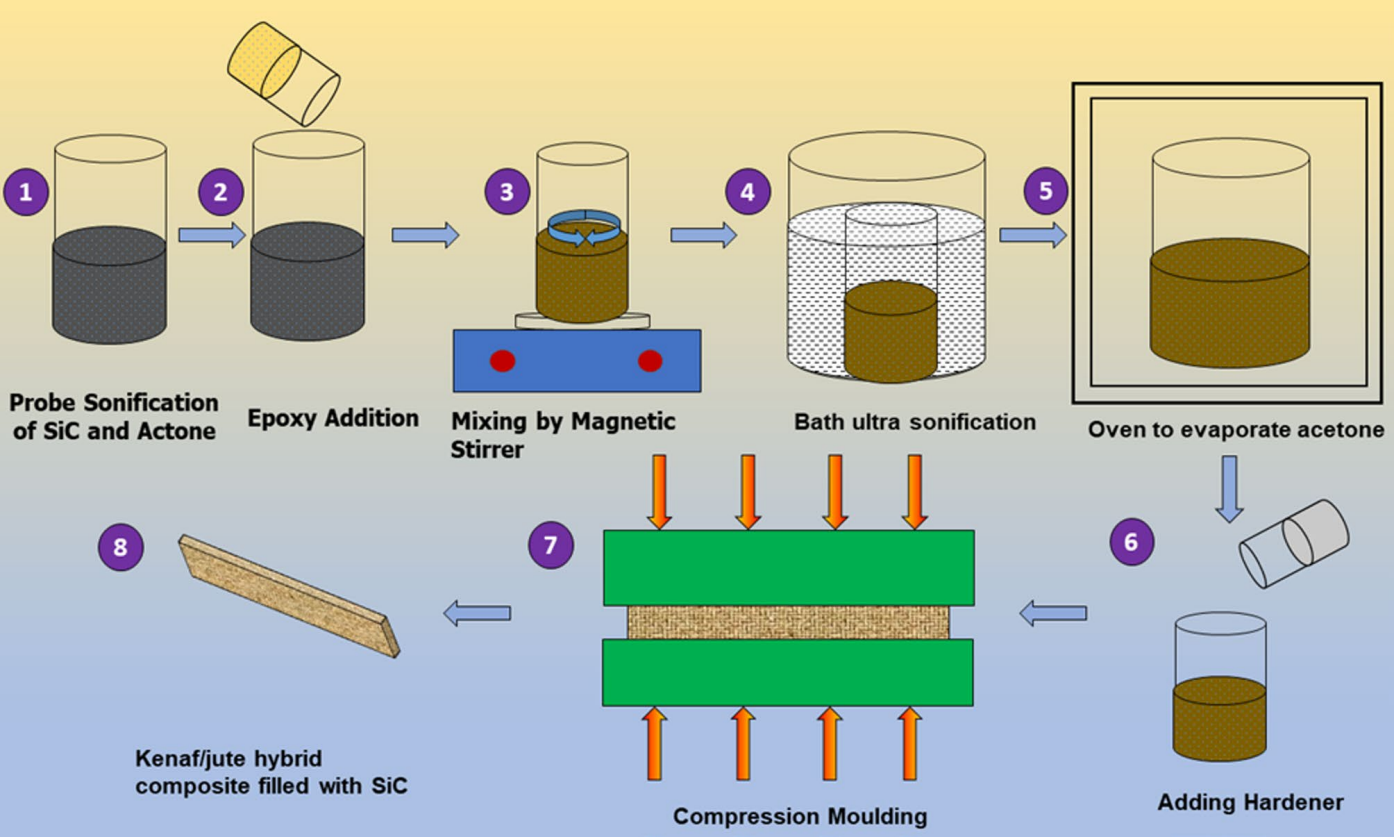
Schematic representation of fabrication process of Kenaf/jute hybrid composite filled with SiC.

The fibers are treated with 5% NaOH by weight to improve adhesion between the fiber and matrix before the fabrication process^39,40^. Silicon carbide is used as the filler material. SiC-filled hybrid composite prepared in three different concentrations by the solution casting method. SiC is infused with DMF and acetone via probe sonicator at 70˚C for 30 min. The required amount of epoxy solution is poured into the resultant mixture of SiC solvent and kept in a magnetic stirrer at 60˚C for 30 min (300 rpm) to ensure the complete vapourization of acetone. To produce a homogenous mixture of epoxy and SiC, ultrasonic waves are used in a bath ultrasonicate at 70˚C for 30 min. The fibers were cut to the right sizes and mixed with an prepared epoxy resin mixture to make a uniform ceramic hybrid material using a compression moulding machine for approximately 45 min at 120 °C for 10 MPa continuous pressure to guarantee adequate compaction. The pressure was then restricted to 6 MPa for approximately 5 min to prevent damage to the laminate prior to post-curing. Silicon spray is used to help remove samples from moulds. Then, it is post-cured in the open atmosphere for a further 24 h at a temperature significantly above that of the ambient (60 °C). Figure 2 depicts the fabrication procedure for the SiC-filled hybrid composite. The fabricated laminated into blocks of 300 mm × 300 mm × 3 mm.

### Characterization of developed hybrid composite

#### Tribology test

**Fig. 3:**
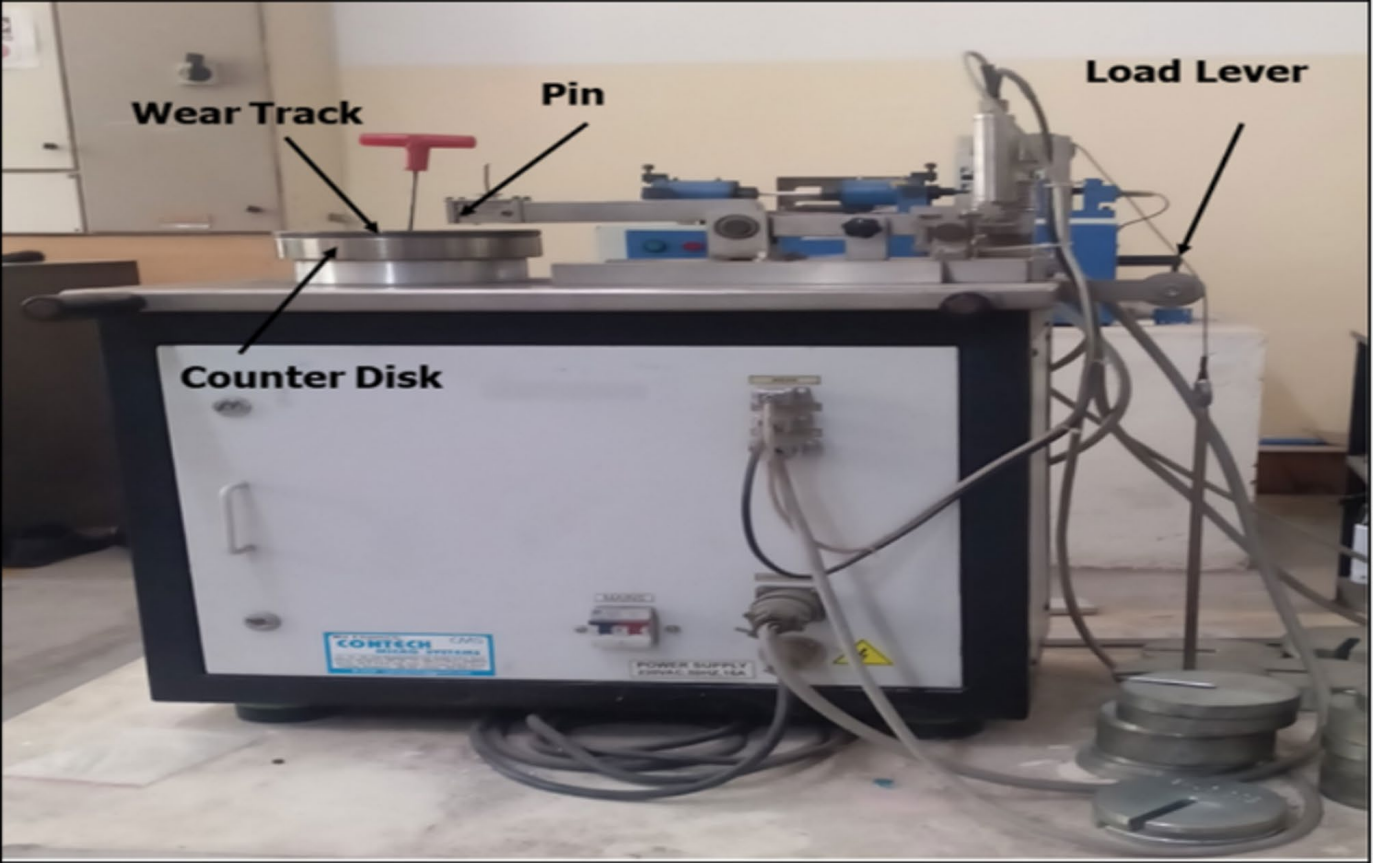
Pin on disk wear testing setup.

The pin-on-disc machine was utilized to conduct the experiments, with a standard AISI D2 steel disc serving as the counter surface. The pin-on-disc experimental setup is illustrated in Fig. 3. The experiments are carried out as per ASTM G99-05. To minimize experimental errors, each sample was tested twice based on the Taguchi orthogonal array. The analysis was based on the average steady-state coefficient of friction (COF), which was calculated according to Eq. 1.

1 $$\text{COF }= \frac{F}{W}$$

Here, the frictional force is denoted as F, load is represented as W.

The volume loss was determined by measuring the weight of the sample before and after the tribological test, as shown in Eq. 2. Subsequently, the SWR is calculated by Eq. 3

2 $${\text{V}}_{{{\text{loss}}}} = \frac{{m_{loss} }}{\rho }$$

3 $$\text{S }= \frac{{V}_{loss}}{W \times L}$$

where m_loss_ is the mass loss (g), V_loss_ is the volume loss (mm^3^), and ρ is the bulk density (g/mm^3^). For Eq. 3, S is the specific wear rate (mm^3^/Nm), and L is the sliding distance (m). A scanning electron microscope is employed to observe and analyze the wear debris area.

#### Water absorption test

The water absorption tests were conducted in accordance with the ASTM D570–98 standard. The size of the sample was 20 × 20 × 10 mm. The samples were initially dried in a hot air oven and weighed to determine their dry weight. They were then submerged in a water bath over a period of ten days with an ambient temperature (32 °C). The wet weight was recorded periodically every 24 h, and the weight gained was determined by subtracting the initial from the final. This process was repeated over the course of ten days, and the water absorption for the samples was calculated using Eq. 4

The calculation of water absorption for the samples was performed using the following Eq. 4.

4 $${\text{Water }}\;{\text{absorption \% }} = { }\left[ {\frac{Wet\;weight - Dry\;weight}{{Dry\;weight}}} \right] \times 100$$

#### Mechanical test

Tensile, flexural, impact, and Shore D hardness tests in both dry and wet environments tested the mechanical characteristics of the created composites. Tensile strength was measuring using computerized Universal Testing Machine (UTM) with a 100KN cell, following ASTM D638 standards. The test was carried out at constant speed of 5 mm/min using rectangular specimens of dimensions 200 × 25 x 4 mm. Every sample underwent three replicas, and the average result was recorded.

Using a 5-mm/min crosshead speed, ASTM D790’s definition of flexural strength matched the same UTM. The flexural specimens measured 150 × 25 × 4 mm. Three repetitions of every sample and the mean reading were taken, as in tensile testing.

ASTM D256 defines impact strength as measured with an Izod Impact Tester. To gauge the energy absorption capacity of the composites, the dimension 60 × 12.5 × 3 mm samples were impacted. Three times the specimens were tested, and an average reading was noted.

Using ASTM D2240 criteria, the Shore D Durometer was used to ascertain Shore D hardness, that is, the resistance of a material to localized plastic deformation by mechanical indentation or abrasion. The test ran for five seconds with an applied load of 49 N. Equation 5, the ratio of applied force (F) to the projected indented surface area (S), allows one to theoretically ascertain the hardness (H) of the composites:

5 $$\text{H }= \frac{F}{S}$$

### Gray relational analysis (GRA)

Gray Relational Analysis (GRA) is a sophisticated data analysis methodology that was originally proposed by Deng Julong in 1982. It is employed to investigate the relationship between two or more variables, and it is particularly employed in situations where complicated parametric analysis involves uncertainty and discrete data. This methodology transforms a multi-response optimization into a single-response process optimization problem by applying either a higher-better or lower-better criterion as an objective.

The primary objective of this study is to optimize the tribological behavior responses, particularly the specific wear rate and coefficient of friction, with a multi-response approach. In order to achieve this, a "lower the better" criterion has been applied to the tribological behavior in multi-response features, thus converting them into a single response. The goal is to decrease the effectiveness of the responses using Eq. 6. In this regard, to minimize the output responses as represented by Eq. (6), the "lower the better" criterion is adopted.

6 $$x_{j} \left( q \right) = \frac{{\max y_{j} \left( q \right) - y_{j} \left( q \right) }}{{\max y_{j} \left( q \right) - min y_{j} \left( q \right) }}\quad \left( {{\text{Lower}}\;{\text{the}}\;{\text{better}}} \right)$$

Likewise, “higher the better” criteria can be employed to maximize the output responses as represented by Eq. (7)

7 $$x_{j} \left( q \right) = \frac{{y_{j} \left( q \right) - min y_{j} \left( q \right) }}{{\max y_{j} \left( q \right) - min y_{j} \left( q \right) }}\quad \left( {{\text{Higher}}\;{\text{the}}\;{\text{better}}} \right)$$

where ‘j’ = no of trials and ‘q’ is the safer operating function, after GRA, ${x}_{j}\left(q\right)$ represents the response data that has been normalized, ${max y}_{j}\left(q\right)$ and min ${y}_{j}\left(q\right)$ are the maximum and minimum from ${y}_{j}\left(q\right)$ responses respectively. A process called normalization reduces variability in tribological behavior, such as specific wear and coefficient of friction. After normalization, the grey relation coefficient (GRC) and grey relation graph (GRG) can be determined. Prior to determining GRG, it is necessary to obtain the grey relational coefficient through Eq. (8).

8 $$\xi_{j} \left( q \right) = \frac{\Delta min + \xi \Delta max}{{\Delta_{0j} + \xi \Delta max}}$$

where $\Delta_{0j}$ = $x_{0} \left( q \right)$ − $x_{j} \left( q \right)$ = Absolute difference between the original sequence $x_{0} \left( q \right)$ and normalized sequence $x_{j} \left( q \right)$, $\Delta max =$ $x_{0} \left( q \right)$ − $x_{j} \left( q \right)$ = large value of $\Delta_{0j}$, $\Delta min =$ $x_{0} \left( q \right)$ − $x_{j} \left( q \right)$ = low value of $\Delta_{0j}$.

In general, In most situations, $\xi_{i}$ is assigned a value of 0.5.

The calculation of GRG involves determining the average value of the grey relational coefficient, which serves to indicate the difference between the reference and comparison. This relationship is determined through the use of the following Eq. (9).

9 $${y}_{i}= \frac{1}{\eta } \Sigma \frac{1}{{y}_{i}^{2}}$$

Here ${y}_{i}$ ranges between 0 and 1, while $\eta$’is the number of outcomes.

The outcome acquired from the experiments and their correlation with actual outcomes demonstrate superior performance compared to other techniques. As a result of the vagueness and uncertainty inherent in experimental results, the GRA method produces imprecision in data due to material inhomogeneity, material architecture, harsh environments and physical variability, and machine error. Therefore, to reduce the uncertainty and improve performance, fuzzy logic is employed in the multi-response optimization process aimed at decreasing the effectiveness of the responses.

### Fuzzy logic approach (FL)

Fuzzy logic is a computational framework that is particularly well-suited for handling problems that involve imprecision, uncertainty, and ambiguity. One of the key advantages of fuzzy logic is its ability to handle problems that are difficult to model, which is crucial in real-world engineering applications^46^. In this context, the integration of artificial intelligence techniques, such as fuzzy logic, can be employed. Fuzzy logic has found wide applications in various fields such as robotics, control systems, aerospace, pattern recognition, data analysis, hybrid systems, and HVAC (heating, ventilation, and air conditioning) control. In the present study, the Mamdani fuzzy inference method is employed owing to its numerous advantages, including the ability to handle uncertainty and imprecision, a superior decision-making process, flexibility to model complex and nonlinear systems, and the capability to integrate human knowledge and expertise into the decision-making process^47^. As a result, fuzzy logic is crucial to evaluate this complex parametric model, which generates imprecise data with uncertainty, which can be resolved by fuzzy logic-based AI techniques, such as neural networks and genetic algorithms, in order to provide more robust and efficient models.

Predicting output responses involves four steps as follows:

- Rule base or knowledge base: Rules and conditions set by humans for the system
- Fuzzification module: Fuzzification transforms crisp inputs, i.e., numerical values, into fuzzy sets. The term crisp inputs refer to exact inputs that are independent variables
- Interference engine: An inference engine determines how closely fuzzy inputs match each rule. These rules are then combined to form the control actions by determining which ones need to be fired.
- Defuzzification module: The defuzzification module transforms fuzzy sets into crisp values obtained by the inference engine.

**Fig. 4:**
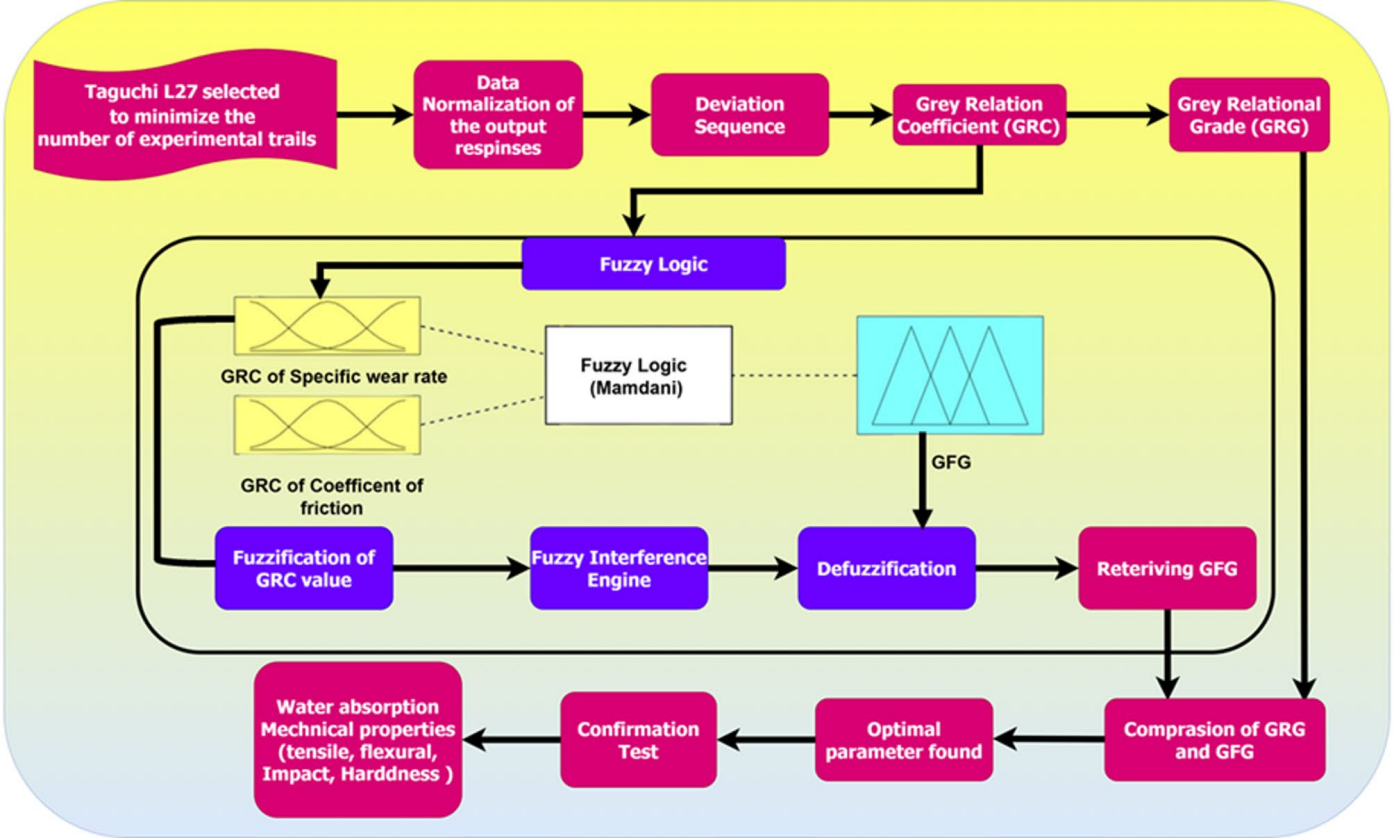
Detailed methodology of grey-fuzzy logic.

A fuzzy inference system (FIS) is employed between the fuzzifier and the defuzzifier to provide a fuzzy interface, and the overall methodological framework of grey-fuzzy logic is illustrated in Fig. 4. FIS is responsible for receiving the fuzzy inputs from the fuzzification module and processing them through a rule base editor. This editor contains a 27 fuzzy set rules, typically expressed as "if–then" statements, which guide the decision-making process.

**Fig. 5:**
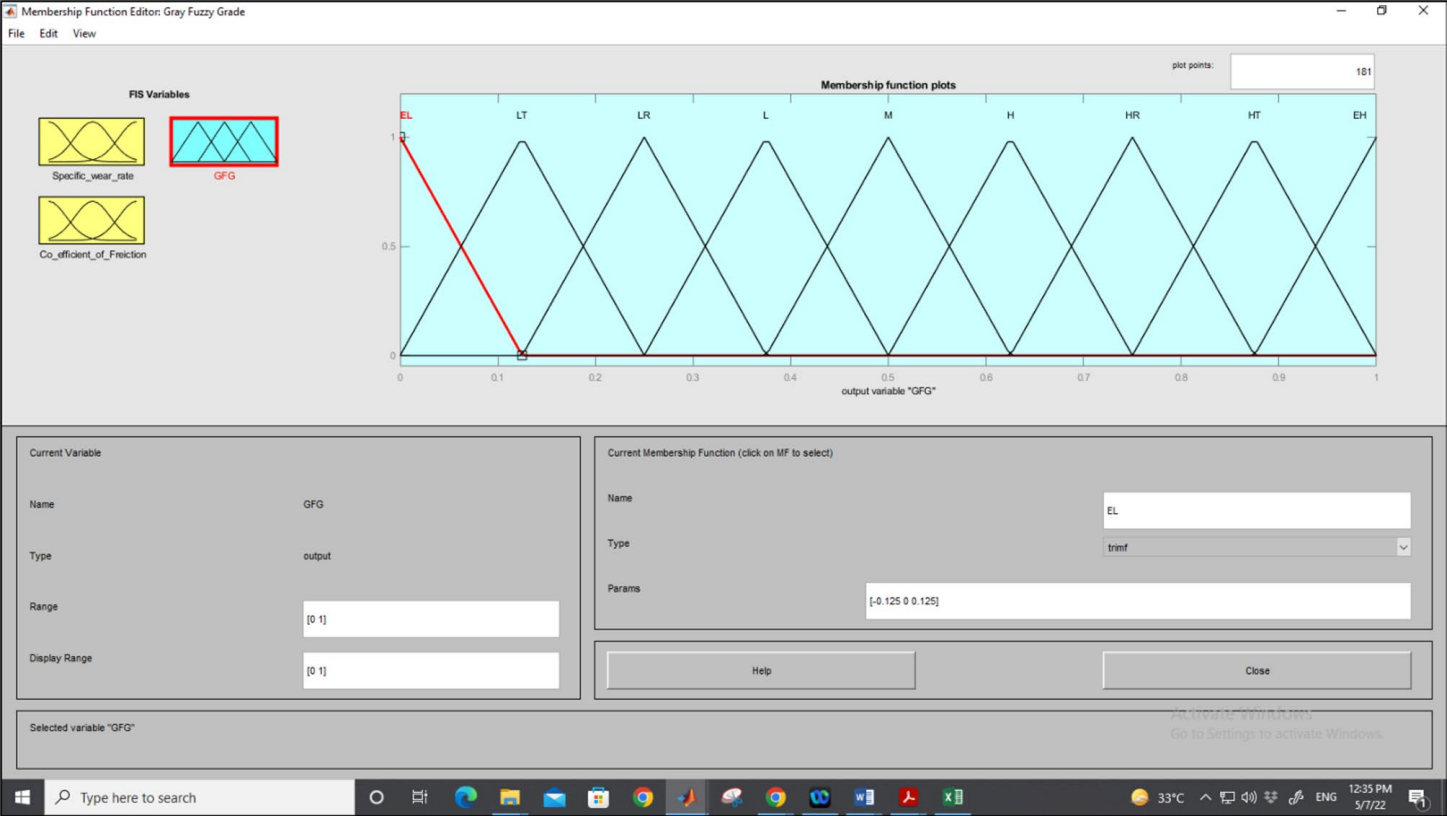
Nine-TMF to transform the fuzzy linguistic variable into a crisp output.

The grey-fuzzy analysis converts grey relational coefficients (GRCs) into fuzzy values. Each input in each fuzzification process is employed using a triangular membership function (TMF), with fuzzy linguistic variable subsets categorized as in the range of small (S), medium (M), and large (L). These subsets correspond to specific segments within the GRC range, which spans from 0 to 1. Finally, the defuzzification module converts the fuzzy output produced by the FIS into a numerical value (crisp output). Figure 5 illustrates the usage of nine TMF to transform the fuzzy linguistic variable into a crisp output.

The results generated by the defuzzification are known as Grey Fuzzy Grades (GFG) and are expressed in nine fuzzy linguistic terms: Extremely Low (EL), Lowest (LT), Lower (LW), Low (L), Medium (M), High (H), Higher (HR), Highest (HT), and Extremely High (EH), as illustrated in Fig. 5. Each of these linguistic terms is associated with a membership function, t₁ and t₂, where t₁ is the grey relational coefficient (GRC) for the specific wear rate, while t₂ is the GRC for the coefficient of friction. The output response variable Y is also expressed as a Grey Fuzzy Grade (GFG) variable.

A total of 27 IF–THEN rule-based fuzzy rules has been defined to describe the correspondence between the input variables (t₁ and t₂) and the resulting output (Y). The rules as follows.

Rule: if condition then “restriction”.

- Rule 1: if t_1_ is A_1_ and t_2_ is B_1_ then Y is C_1_; else
- Rule 2: if t_2_ is A_2_ and t_2_ is B_2_ then Y is C_2_; else
- Rule 3: if t_3_ is A_3_ and t_3_ is B_3_ then Y is C_3_; else
- ………………………………………………
- Rule 27: if t_n_ is A_n_ and t_n_ is B_n_ then Y is C_n_ else.

These rules improve the fuzzy inference process by linking pairs of input linguistic variables to their corresponding output fuzzy sets. The inference engine utilizes this rule base to process the input fuzzy then employs this rule base to process the input fuzzy sets and generate an output fuzzy set. This fuzzy output is then defuzzified, which quantitatively defines the output of the system. The independent variables are denoted by t_1_, t_2_, and t_3_. The various fuzzy subsets, Ai and Bi, are described by their respective membership functions, µAi and µBi. These IF–THEN rules are formulated to compute the overall GFG (multi-response output) using the max–min operation with linguistic variable subsets along the TMF. This process is mathematically expressed as Eq. 10.

10 $$\mu {\text{C}}_{0} \left( {{\text{Y}}_{0} } \right) = {\text{Max}}\left[ {{\text{Min}}_{{\text{u}}} \left\{ {\mu {\text{A}}_{1} {\text{t}}_{1} \left( {{\text{t}}_{1} } \right),\mu {\text{B}}_{2} {\text{t}}_{2} \left( {{\text{t}}_{2} } \right) \ldots \ldots \ldots \ldots \ldots \mu {\text{Ct}}_{{\text{n}}} \left( {{\text{t}}_{{\text{n}}} } \right)} \right\}} \right]$$

The defuzzifier is responsible for converting the fuzzy inference output, represented by μC_i_(t_i_), into a numerical, non-fuzzy value using Eq. 11. This value corresponds to the Grey Fuzzy Grade (GFG)^33^ output Yy

11 $${y}^{y}=\frac{\sum y\mu {C}_{0}\left({y}_{0}\right)}{\sum \mu {C}_{0}\left({y}_{0}\right)}$$

The GFG values are evaluated in comparison to the GRG and it should be noted that the GFG value should ideally be lesser than the Grey Relational Grade (GRG) that is achieved by the Grey Fuzzy Approach (GFA). The Centroid of Area (COA) technique determines the centroid of the fuzzy output that is processed by the fuzzy inference system (FIS). This defuzzification technique utilizes the TMF to convert the multi-response output into precise numerical value.

#### Confirmation test

A confirmation test was applied to provide validation for the decrease in multi-response outputs to predict the peak performance of particulate natural hybrid composites. The result of the confirmation test was verified using the Grey Fuzzy Grade (GFG) value. For the computation of the GFG under optimal conditions, Eq. (12) was utilized.

12 $${y}_{predicted}= {y}^{y}+ \sum_{i=1}^{n}{y}_{j- } {y}^{y}$$

This equation has several variables: y_j_ represents the overall average of the GFG value at the optimal point, yʸ represents the average GFG value, and η represents the number of factor conditions.

## Results and discussion

### Gray relation analysis

The GRG and GFG for experimental trials, their rank, and comparison are listed in Table 4. This provides the experimental data arranged in line with the Design of Experiments (DoE) approach. Equation (6) defines a "lower-the-better" criterion that was used pre-processing all output response data. The Grey Relational Coefficients (GRCs) were then computed for every 27 experimental runs using a specified reference sequence. Especially, the 19th experimental trial produced the best GRC value of 0.8045, so indicating better performance among all the tested combinations.

**Table 4:** GRG and GFG for experimental trials, their rank and comparison.

Exp. no	Output responses		Gray relational coefficient (GRC)		Gray relational grade (GRG)	Rank	Gray fuzzy grade (GFG)	Rank	% Error
	SWR (× 10^–5^ mm^3^/Nm)	COF	SWR	COF					
			Ideal sequence						
			1.000	1.000					
1	3.942	0.372	0.4691	0.6295	0.5493	9	0.538	9	2.06488
2	4.196	0.444	0.4208	0.5433	0.4820	13	0.479	13	0.632636
3	4.843	0.619	0.3333	0.4076	0.3704	27	0.365	27	1.471098
4	3.968	0.514	0.4637	0.4794	0.4716	15	0.462	15	2.026919
5	4.02	0.634	0.4532	0.3990	0.4261	21	0.417	21	2.133051
6	4.185	0.715	0.4227	0.3584	0.3906	26	0.382	26	2.193753
7	3.868	0.642	0.4854	0.3946	0.4400	17	0.431	17	2.044303
8	3.954	0.775	0.4666	0.3333	0.4000	25	0.392	25	1.992654
9	4.01	0.724	0.4551	0.3544	0.4048	23	0.402	23	0.68948
10	3.746	0.354	0.5148	0.6556	0.5852	7	0.575	7	1.737026
11	4.07	0.338	0.4435	0.6806	0.5620	8	0.559	8	0.537607
12	4.394	0.604	0.3895	0.4165	0.4030	24	0.398	24	1.241844
13	3.907	0.524	0.4767	0.4715	0.4741	14	0.465	14	1.918795
14	3.895	0.643	0.4793	0.3941	0.4367	18	0.428	19	1.991132
15	3.984	0.674	0.4604	0.3779	0.4191	22	0.415	22	0.988962
16	3.814	0.693	0.4980	0.3686	0.4333	19	0.429	18	0.99068
17	3.855	0.644	0.4884	0.3935	0.4409	16	0.435	16	1.346217
18	3.957	0.655	0.4660	0.3876	0.4268	20	0.421	20	1.361522
**19**	**3.434**	**0.204**	**0.6090**	**1.0000**	**0.8045**	**1**	**0.801**	**1**	**0.437541**
20	3.758	0.234	0.5117	0.9049	0.7083	3	0.702	2	0.891327
21	4.01	0.376	0.4551	0.6240	0.5396	10	0.531	10	1.592623
22	3.564	0.334	0.5659	0.6871	0.6265	5	0.620	5	1.036056
23	3.594	0.472	0.5567	0.5158	0.5363	11	0.531	10	0.984241
24	3.668	0.547	0.5355	0.4543	0.4949	12	0.490	12	0.9841
25	3.392	0.368	0.6244	0.6352	0.6298	4	0.621	4	1.396167
26	3.391	0.374	0.6248	0.6268	0.6258	6	0.618	6	1.246054
27	2.768	0.482	1.0000	0.5067	0.7533	2	0.651	3	13.58339

Significant values are in [bold].

### Gray fuzzy analysis

**Fig. 6:**
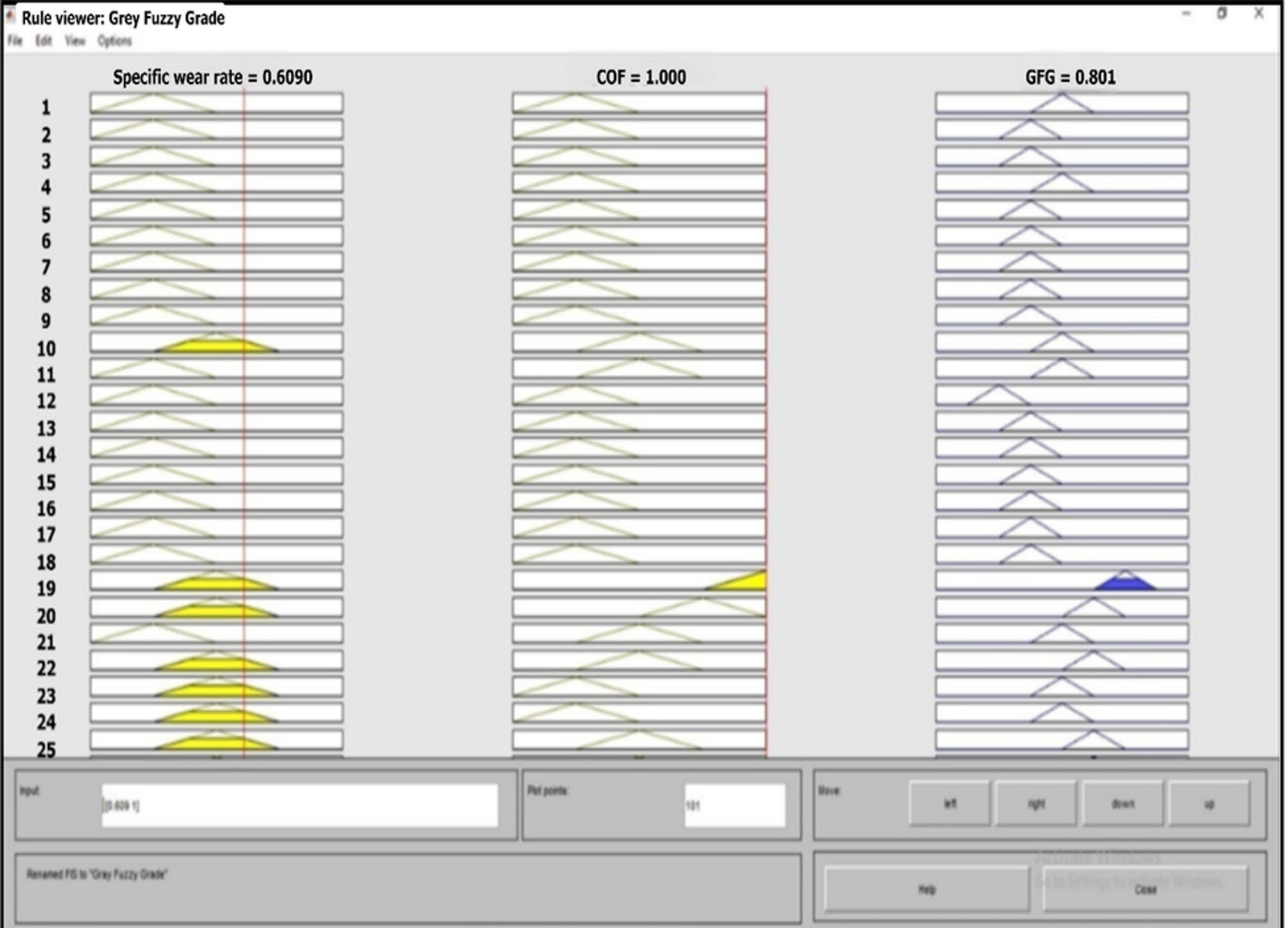
GFG output response based on fuzzy rules.

The Grey Fuzzy Grade (GFG) values for all experiments performed were calculated using MATLAB R2019a, from the fuzzy inference system environment. The best crisp output value of 0.801 was achieved during the nineteenth trial of the experiment, as confirmed by the rule viewer of the fuzzy inference engine. As seen from Fig. 6, the corresponding Grey Relational Coefficients (GRCs) for the specific wear rate and the coefficient of friction were 0.6090 and 1.000, respectively. The crisp outputs of all 27 experiments were derived using the fuzzy rule base editor and tabulated systematically in Table 4.

**Fig. 7:**
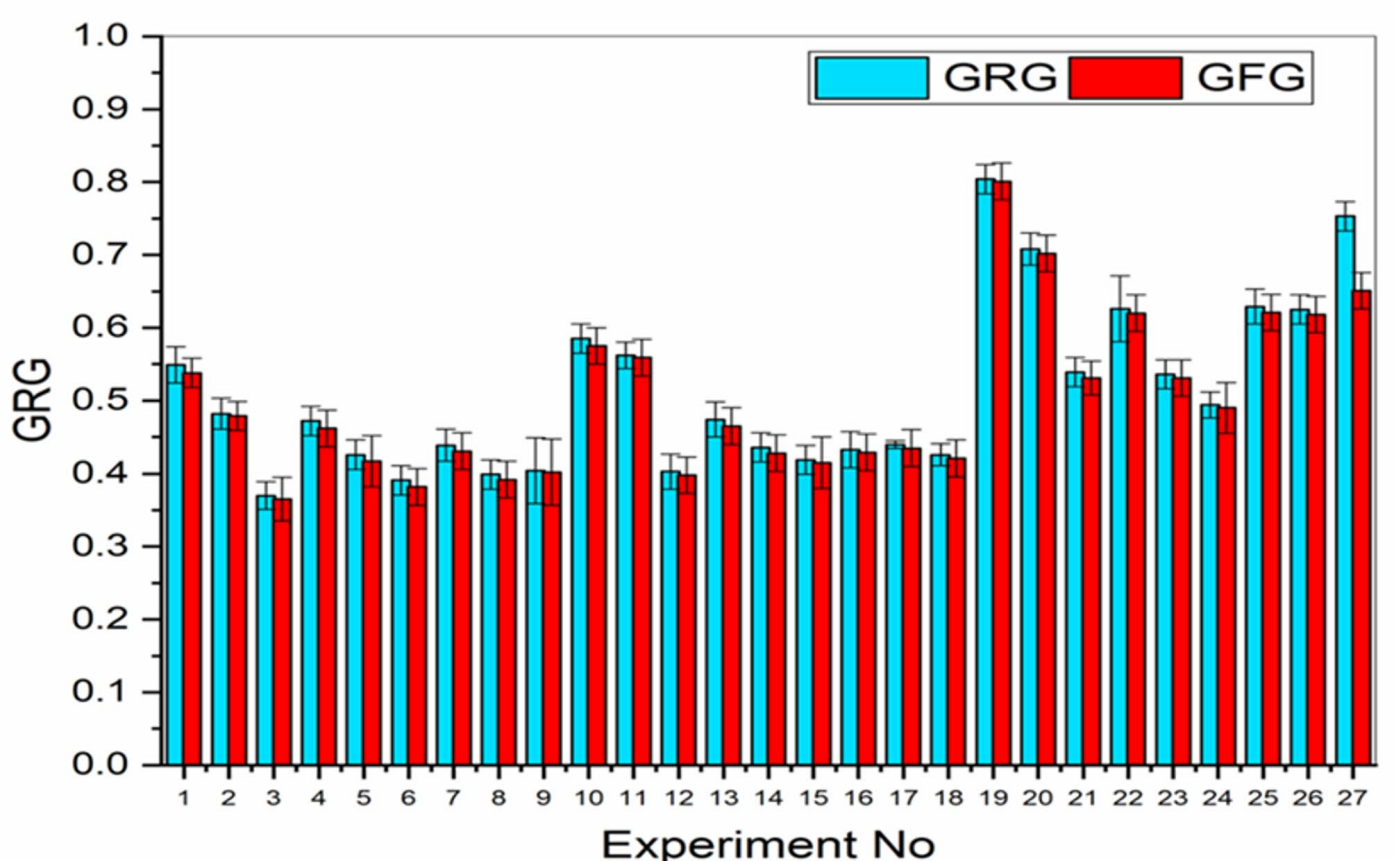
Differences between GRG and GFG.

As shown in Fig. 7, a comparative study of the Grey Relational Grade (GRG) and the Grey Fuzzy Grade (GFG) indicates a decrease in output uncertainty by means of the fuzzy logic-based GFG technique. Especially, a 1.83% decrease in GFG compared to the general response of GRG shows the greater dependability and accuracy of the fuzzy method. In addition, Table 4 shows negligible variation in GFG values in all the experiments performed, thus reflecting uniform system performance.

**Fig. 8:**
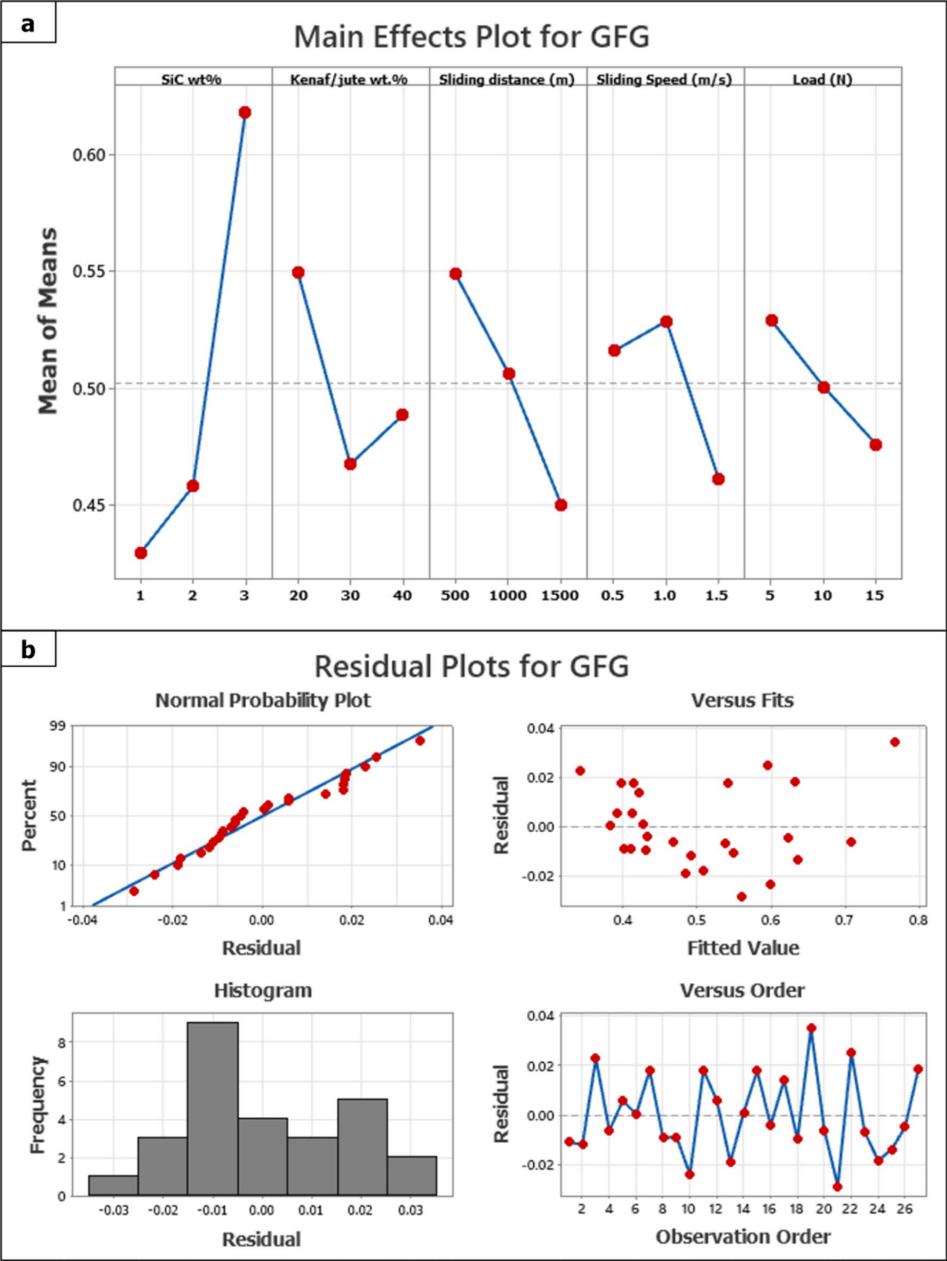
(**a**) Main effect plot of GFG, (**b**) response plot graph for GFG.

Figure 8a shows the main effect plot of GFG, and Fig. 8b shows the response plot graph for GFG. In order to analyze the influence of individual process parameters, a main effect plot and a residual analysis of the GFG responses were conducted with Minitab statistical software. The response table for the Gray Fuzzy grade (Lower is better) is listed in Table 5. Based on the factor-level analysis, the optimal levels of the parameters were found as A_1_B_2_C_3_D_3_E_3_, which translates to 1 wt% Gr/SiC, 30 wt% reinforcement, 1500 m sliding distance, 1.5 m/s sliding speed, and 15 N applied load. In addition to that, it was discovered that the delta value that separates the maximum from the minimum GFG responses. With the highest GFG value of 0.801, the nineteenth trial exhibited the best multi-response performance, thus confirming the efficiency of the fuzzy inference-based optimization system. The corresponding composite configuration that exhibit significantly improved wear resistance and stable fractional behavior under milder condition, which is suitable is light duty tribological applications. Furthermore, the obtained Grey-Fuzzy based multi-response framework in our present study identified optimal configurations with GFG value above 0.8, which is comparable to the range of 0.78–0.85 observed in similar multi-responses optimization^48–50^. This comparative alignment strengthens the suitability of our approach for modelling and optimizing wear performance under milder load conditions, while offering a simplified yet but effective way to make decision making.

**Table 5:** Response table for gray fuzzy grade (lower is better).

Level	SiC wt%	Kenaf/jute wt.%	Sliding distance (m)	Sliding speed (m/s)	Load (N)
1	0.4298	0.5498	0.5491	0.5162	0.5293
2	0.4583	0.4678	0.5068	0.5290	0.5009
3	0.6183	0.4889	0.4506	0.4612	0.4762
Delta	0.1886	0.0820	0.0986	0.0678	0.0531
Rank	1	3	2	4	5

### Analysis of variance (ANOVA)

Analysis of Variance (ANOVA) was applied to ascertain the statistical significance and fitness of the established model. ANOVA is a strong statistical method used to analyze individual and combined influences of control factors on output responses and hence ascertain the most influential parameters. The ANOVA results, as presented in Table 6, indicate that the weight percentage of silicon carbide (SiC wt%) is the most influential factor on the response variables. This is attested by a P-value of 0.000, which is far below the significance level of 0.05, showing the high level of statistical significance. Additionally, the corresponding F-value of 22.21 confirms that SiC weight percentage has a significant impact on the fuzzy system response. The coefficient of determination (R^2^), a statistical measure of the percentage of variance in a variable that can be accounted for by independent variables, was found to be 0.944. These R^2^ value confirmed the statistical significance of P < 0.05 consistent with findings by others^21,51,52^, validating the model’s reliability in tribology response prediction.

**Table 6:** Analysis of variance (ANOVA) for grey fuzzy grade.

Source	DF	Adj SS	Adj MS	F-Value	P-value	Remarks
SiC wt%	1	0.008506	0.008506	7.29	0.016	Significant
Kenaf/jute wt.%	1	0.022062	0.022062	18.91	0.001	Significant
Sliding distance (m)	1	0.013535	0.013535	11.60	0.004	Significant
Sliding Speed (m/s)	1	0.003704	0.003704	3.17	0.095	Significant
Load (N)	1	0.000858	0.000858	0.73	0.405	Insignificant
SiC wt%*SiC wt%	1	0.025916	0.025916	22.21	0.000	Significant
Kenaf/jute wt.%*Kenaf/jute wt.%	1	0.018888	0.018888	16.19	0.001	Significant
SiC wt%*Kenaf/jute wt.%	1	0.000014	0.000014	0.01	0.914	Insignificant
Kenaf/jute wt.%*Sliding distance (m)	1	0.009734	0.009734	8.34	0.011	Significant
Sliding distance (m)*Sliding Speed (m/s)	1	0.004421	0.004421	3.79	0.071	Insignificant
Sliding Speed (m/s)*Load (N)	1	0.000289	0.000289	0.25	0.626	Insignificant
Error	15	0.017504	0.001167			
Total	26	0.316115				

R-Sq = 94.46, R-sq (adj) = 90.40%

### Regression analysis

The grey fuzzy grade can be predicted based on predetermined levels of factors through the utilization of regression analysis. This analysis employs a quadratic polynomial regression equation to represent the relationship between the factors and the grey fuzzy grade.

$$\begin{aligned} {\text{GFG}} = & {1}.{665} - 0.{\text{1719 A}} - 0.0{662} {\text{B}} - 0.000{\text{428 C}} \\ + & 0.{2}0{7} {\text{D }} + 0.00{\text{552 E }} + 0.0{657} {\text{A}}*{\text{A}} \\ + & 0.000{742} \left( {{\text{B}}*{\text{B}}} \right) \, + 0.000{1}0{8} \left( {{\text{A}}*{\text{B}}} \right) \\ + & 0.0000{16} \left( {{\text{B}}*{\text{C}}} \right) \\ - & 0.000{154} \left( {{\text{C}}*{\text{D}}} \right) \\ - & 0.00{278} \left( {{\text{D}}*{\text{E}}} \right) \\ \end{aligned}$$

The residual plots are examined to assess the adequacy and goodness fit of integrity of the proposed model, as shown in Fig. 8b. The residual represents the disparity between the estimated value and the fitted value, capturing the variability therein. In general, residual plots are a graphical way to assess whether a model fits the data well. Typically, a probability plot is characterized by each response that are is aligned tightly along a straight line^53,54^. Furthermore, a high degree of closeness was evident from the versus fits analysis of the constructed regression model. Histograms and residuals versus order plots visualize the distribution of residuals against the order of data points and reveal that normally distributed on a specific side. Table 7 presents comparable studies that employed a multi-response framework to optimize the friction and wear.

**Table 7:** Comparable studies employed multi-response framework to optimize the friction and wear.

Composite type	Methods and metrics	Key outcomes	Ref
padystraw + pineappleleaf polyester composite	Machine learning + RSM	Regression predicted the validation of the model up to 0.999, demonstrating the excellent predictive performance	^55^
Flax + Graphene	Taguchi-GRA hybrid model	Muliti-response optimization with high GRG value with excellent predictive R^2^ = 98.30% & R^2^adj = 93.22%, indicating robustness of model	^56^
Glass epoxy	Taguchi-GRA hybrid model	Regression model reported predictive high R^2^ values were 0.8095 (COF) and 0.5972 (wear volume), confirming the robustness of model	^57^
Polytetrafluoroethylene Matrix Composites	GRG hybrid model support vector regression (SVR), SVR-PSO, and SVR- Harris hawks optimization algorithm (HHO),	GRG hybrid models, SVR, SVR-PSO, and SVR-HHO, demonstrated improved predictive accuracy with R^2^ values of 0.5353, 0.7059, and 0.8611, respectively	^58^
Sisal + Banana + Flyash	Taguchi-GRA hybrid model	Multi-response optimization with high GRG and attained optimal performance	^41^
Abaca fiber + Rud Mud filler	RSM-Fuzzy Logic hybrid model	Predicted SWR and COF with R^2^ values of 0.952, indicating robustness of the model	^35^
Glass fiber reinforced polyester	ANN based Fuzzy inference system (FIS)	Predicted the tribological behavior and achieved R^2^ = 0.964, indicating excellent model accuracy	^59^
Kenaf + Jute + 1%SiC	Grey-Fuzzy model	Predicted SWR and COF with achieved R^2^ = 0.945, indicating excellent reliability of the model	the present study

### Confirmation result

**Fig. 9:**
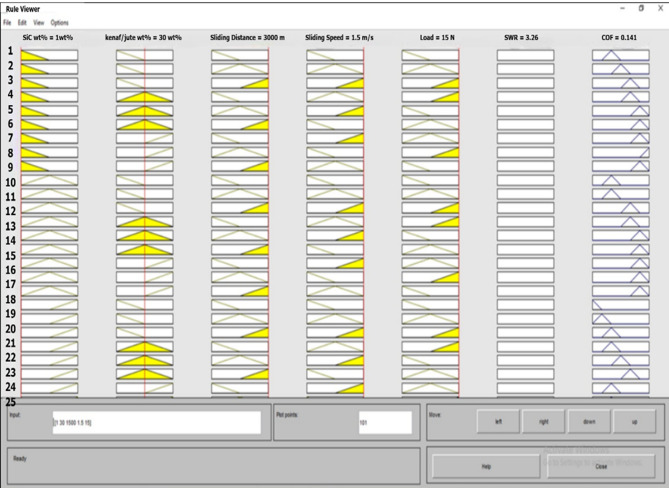
Prediction of optimized combination response.

The research proves that the suggested Taguchi-based Grey Fuzzy Optimization method successfully predicted the best wear conditions with an error range of 0% to 5%. The proposed method can solve multi-response optimization problems efficiently, hence saving experimental cost and time as well as improving the predictive model robustness. The experimental Grey Fuzzy Grade (GFG) values obtained from the trial runs were extremely consistent with the predicted GFG values calculated from the fuzzy inference system. A comparison of the experimental and predicted values of specific wear rate and coefficient of friction is given in Table 8, and it was noted that there is a good correlation between the two results. Similar trend has been reported on either natural or synthetic by Subramanyam et al.^60^, Nayak SK et al.^61^ using computational techniques for Areca Sheath/epoxy composites and waste marble dust filled glass fiber. Furthermore, miniappan PK et al.^62^ using different fillers in areca fiber and similar results also have been observed. The high correlation is visually supported in Fig. 9, which presents the prediction of the optimized combination response and indicates the precision and efficacy of the model in predicting tribological behavior. Additionally, the rule viewer in provides a graphical overview of the response of the fuzzy inference system to fine input parameters and, therefore, contributes to greater insight into the model’s decision-making process. The comparative analysis indicates that the new methodology outperforms conventional methods in terms of predictive accuracy. Notably, the difference between the theoretical and experimentally calculated values was below 5%, which is within the limits of acceptability in engineering, and thus corroborated the validity and applicability of the model.

**Table 8:** Initial and optimal parameter setting level.

Initial parameter setting		Optimal parameter settings		Error %
		Prediction	Experimental	
Level	A_1_B_1_C_1_D_1_E_1_	A_1_B_2_C_3_D_3_E_3_	A_1_B_2_C_3_D_3_E_3_	
Specific Wear Rate (× 10^–5^ mm^3^/N-m)	3.942	3.26	3.338	2.33
Coefficient of friction	0.372	0.141	0.154	8.4
GFG		0.854	0.826	2.8

### Worn surface morphology

**Fig. 10:**
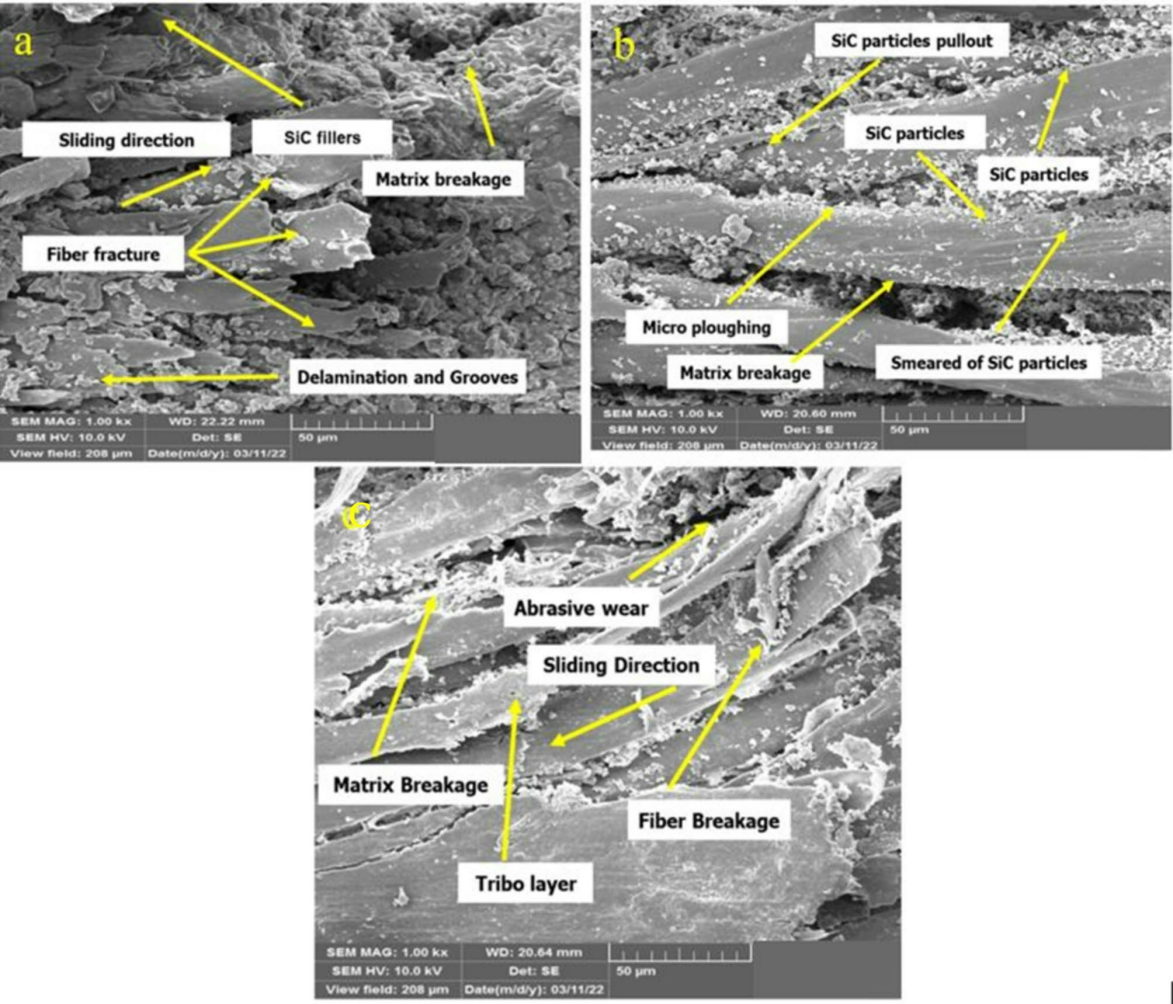
Worn Surface morphology (**a**) 1%SiC, 20 wt.% KJ reinforcement, 500 m Sliding Distance, 0.5 m/s Sliding Speed, 5 N Load (**b**) 3 wt % SiC, 20% reinforcement, 1500 m Sliding distance, 1.5 m/s sliding speed, 15 N load (**c**) 1 wt %SiC, 30% reinforcement, 1500 m sliding distance, 1.5 m/s sliding speed, 15 N load.

The wear morphology of worn-out surfaces of the particulate hybrid composites was examined with aid of a scanning electron microscope (SEM) at different harsh adverse conditions particularly high wear with low COF, high wear with high COF and low wear and low COF and displayed in Fig. 10a–c. Surface degradation mechanisms related with increased friction and wear responses were characterized using higher magnification imaging.

#### High wear–low COF

Corresponding to the high wear–low COF condition, Fig. 10a shows microstructural characteristics including fine furrows, micro-scratches, fiber pull-out, matrix cracking, fiber debris, and ploughing marks on the worn surface. Based on above mechanism, the present study clearly visible are indicators of elastic deformation of fibers and brittle fracture of SiC particulates. This is because the inappropriate contact at the counter interface might be associated with inconsistent material constituents with low load, low sliding speed, and low sliding distance, resulting in a small contact area that would cause the hindering of the synergistic effect of fiber and SiC filler and inadequate formation of tribofilm^8,63^.

#### High wear–high COF

On the other hand, improved interfacial contact generates significant thermal and mechanical stresses under high load, sliding speed, and sliding distance Fig. 10b. Especially at the fiber-matrix interface, these stresses encourage matrix softening, which causes exfoliation and generates fine wear debris. The Ploughing Effect, a dominant abrasive, arises when SiC hard particles penetrate the softer polymer matrix, producing deep grooves or scratches, especially under higher loads, leading to increased material removal. This phenomenon is well-documented in previous studies^64,65^.

In parallel, rough Tribo-film formation occurs due to agglomeration or fiber fragments under high stress or moisture exposure. This leads to increased surface roughness and unstable wear behavior, as noted in previous studies^66^. Elevated shear forces intensify the fiber degradation and encourage irregular triboflim development, contributing to the viscoelastic–plastic transition behavior of the polymer matrix. Moreover, under these extreme conditions, too high SiC loading can cause particle agglomeration and consequent detachment from interfacial shear stress accumulation^67,68^.

#### Low wear and low COF

Under rather mild wear conditions, Fig. 10c, the surface morphology shows limited wear debris, a more homogeneous wear track with parallel ploughing lines, and smooth surface features matched with the sliding direction. These characteristics correlated with a low specific wear rate (3.33 × 10⁻^5^ mm^3^/N·m) and COF 0.154 and a high GFG value of 0.826, reflecting the 19th trial. This value indicated an enhanced surface protection and energy dissipation under stable conditions. These enhanced contact homogeneity helps the matrix to undergo localized thermal softening, allowing stable exposure of reinforcing fibers and encouraging the development of a continuous and adherent tribofilm.

Furthermore, the synergistic effect between SiC and natural fiber significantly contributed to improved wear resistance by increasing surface hardness, promoting uniform load distribution, enhancing energy dissipation and providing crack arresting mechanism.^69,70^.

Furthermore, SiC fillers played a crucial role in debris encapsulation and in encouraging a transition from sliding to rolling wear, thereby reducing material loss. Similarly, Yallew et al.^71^ proved that incorporating jute fibers within the polypropylene matrix caused an increase of 65% in specific wear and an increase of 3.5–4.5% in the coefficient of friction, confirming the existence of reinforcement and wear trade-off. These findings are in also agreement with previously reported studies on particulate hybrid composites reinforced with natural fibers and SiC, including Areca/Tamarind/SiC^5^, Areca husk/SiC^4^, Basalt/SiC^7^, Oil-palm/Kenaf^70^, Kenaf/Banana^72^, Jute/Coir^73^, Basalt/Aramid^30^, Bagasse/Kevlar^74^, SiC–graphite–butadiene rubber^75^, and Sisal/Banana/Bagasse^76^. By means of tribological synergy, the combined reinforcement effects of fiber and particulate ceramic fillers help to lower friction, minimize ploughing, and improve wear resistance.

Comparatively to the optimizied condition, the wear rate and COF under the high wear–low COF and high wear–high COF conditions raised by 15.32% and 58.60%, respectively, and by 16.75% and 59.04%, respectively. This improvement in tribological performance is mostly related to the capacity of SiC particles to occupy micro-defects inside the epoxy matrix, thereby supporting the development of a strong and durable tribofilm.

### Water absorption and post-mechanical properties

Natural fiber-reinforced composites have the inherent tendency to show hydrophilic behavior owing to the availability of hydroxyl groups in the cellulose molecule, making them prone to moisture absorption in high-humidity or aqueous environments. The goal of this work was, therefore, to optimize the wear resistance of a silicon carbide (SiC)-filled kenaf/jute particulate hybrid composite for potential applications in dry and moisture-rich environments. In view of this, the performance of the composite, its major mechanical properties like tensile strength, flexural strength, and impact resistance were tested prior to and following water absorption.

**Fig. 11:**
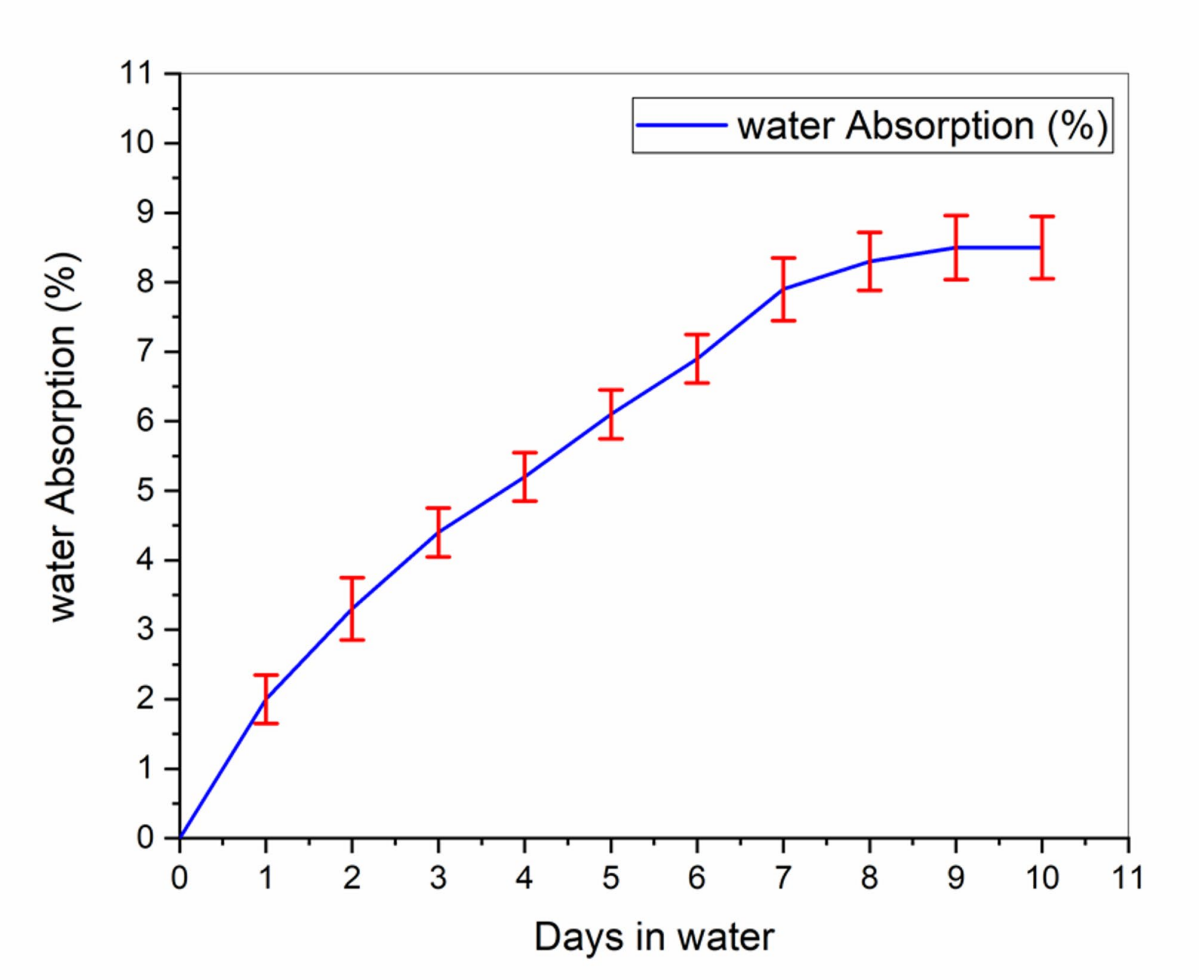
water absorption beaviour of 1wt%SiC 30 wt% kenaf/jute.

Fiber swelling, caused by water absorption, produced micro-cracks at the matrix–fiber interface as a result of differential expansion and matrix debonding^77^. The water uptake behavior of the optimally designed composite structure is shown in Fig. 11. Water absorption percentage was calculated from Eq. (4) and plotted versus immersion time. The findings demonstrated an increase in water uptake as a function of time, achieving the maximum of 8.4% for the optimally designed hybrid system, far below the widely accepted 10% stability limit in polymer composites. The lowest absorption, as observed after 48 h of soaking, was 2.39%.

**Fig. 12:**
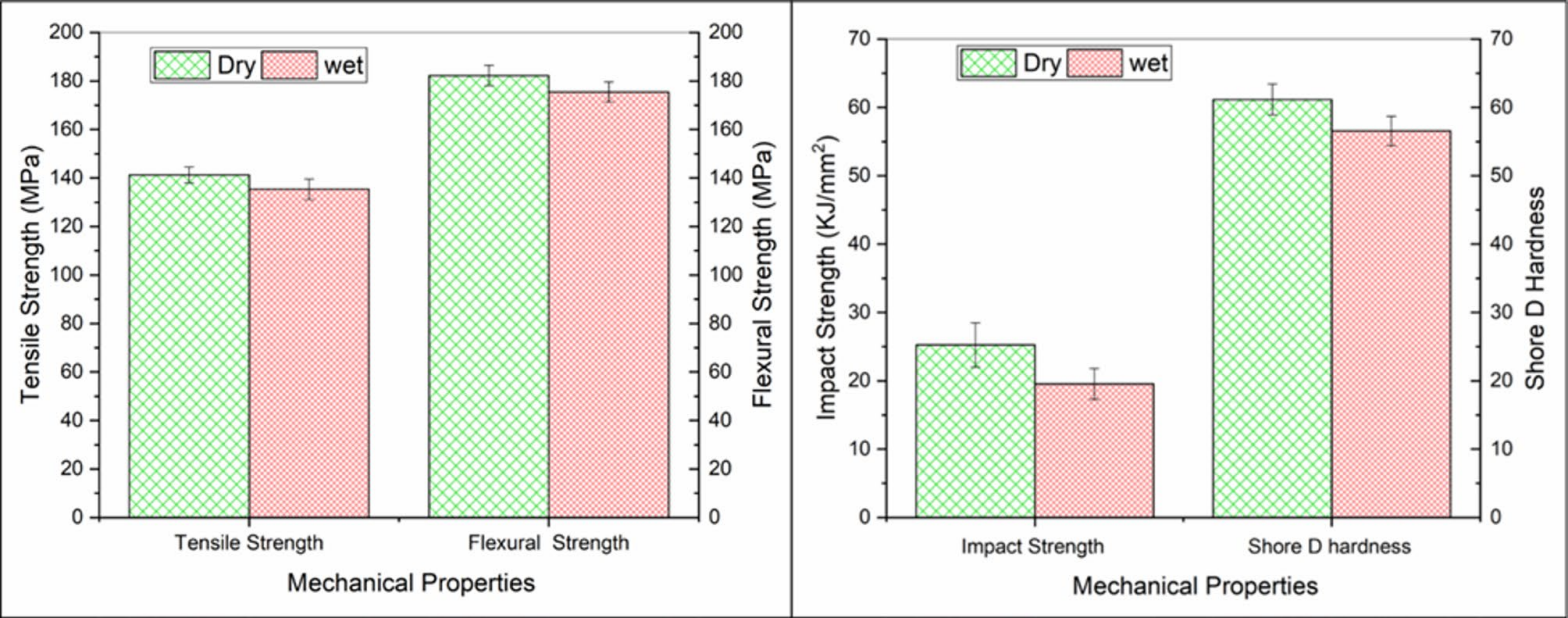
Mechanical beaviour of 1wt%SiC 30 wt% kenaf/jute.

Comparing with a control kenaf/jute composite with 30 wt.% loading of kenaf fibers, the optimized hybrid system exhibited much lower moisture uptake, and the water absorption value was 7.5%^78^. However, jute fiber-reinforced polyethylene composites with comparable fiber content (30 wt.%) have exhibited much lower water absorption values, around 1.2%. This difference emphasizes the higher hydrophilicity of kenaf fibers compared to jute, validating that hybridizing with jute and adding SiC fillers is able to efficiently counteract the tendencies of water absorption by natural fiber composites^79^ (Fig. 12).

The current investigation exhibited moisture absorption behavior consistent with the findings from previous studies on particulate hybrid composite, such as jute/SiC^80^, banana/sisal^81^, bamboo/jute/glass^82^, hemp/jute^83^, flax/PLA^84^, kenaf/Kevlar^85^, and flax/hemp^86^. In all these hybrid composites, characteristics trend of initial rapid water uptake was observed, followed by a gradual reduction in absorption rate, ultimately approaching saturation over prolonged immersion duration.

Furthermore, mechanical properties, including tensile, flexural, and impact tests was conducted between dry and wet samples of particulate hybrid composites. Due to the limited literature on the optimized combination of moisture affected hybrid composites, the study fills the critical gap.

The experimental results indicated that the mechanical properties decreased by 10% after the water absorption test, as shown in Fig. 11. This decrement was due to water molecules attacking the interphase for a prolonged period, resulting in the spiking and swelling of the interphase region, making it weaker^28^. The swelling of the fibers also changes their dimensions, causing microcracks to appear on the matrix surface. Longer immersion times allowed larger water molecules to penetrate into the interphase of the composite, separating the fibers and matrix. The adhesion between fibers and matrix could be one of the primary factors determining the mechanical properties of a composite^87^.

With increasing immersion time, the composite’s mechanical integrity continued to deteriorate. Nevertheless, the present particulate hybrid composite demonstrated a tensile strength of 141.25 MPa compared to the conventional long kenaf fiber composite (30 wt%), which typically exhibited a tensile strength around 39.2 MPa. These enhancements underscore the efficiency of hybridization and filler incorporation^88,89^.

In terms of relative performance improvements, the tensile strength of the present particulate hybrid composite surpassed that of kenaf/sisal^88^, kenaf/jute^90^, kenaf/hemp^78^, and hemp/jute^83^ by 59.09%, 36.99%, 54.45%, and 53.52%. Corresponding improvements in flexural strength was observed at 59.09%, 47.87%, 50.17%, and 54.45% for optimized 30 wt% combination.

The higher swelling effect on impact resistance and hardness may be caused by capillary cracks and continuous attack of water molecules at the interphase, leading to debonding of fiber and matrix and microcracks on the surface. It has been found that hybridizing kenaf and jute may contribute to the composite’s strengthening effect and increase its water resistance, while SiC filler possesses better wear resistance. The data presented in Singh MK et al.^91^ indicate that a random kenaf composite with 30 wt.% (61.45 MPa) provides more strength than a pure random jute composite with 49.75 MPa, while a particulate hybrid random composite with 63.36 MPa offers a positive hybridization effect. On the other hand, the flexural strength of random kenaf composite with 30 wt.% (54.33 MPa) provides more strength than a pure random jute composite with 50.13 MPa, while a particulate hybrid random composite with 181.5 MPa. Furthermore, mechanical properties of composite directly influence tribological behavior of composite by determining the material resistant to deformation. In general, increased the hardness generally leads to reduced material removal, thereby lowering the wear rate. Similarly, the hardness of random kenaf composite with 30 wt.% (58.7) provides more hardness than a pure random jute composite with 53.7 MPa, while a particulate hybrid random composite with 61.2. The presence of 1 wt% SiC contributes resistance to micro cutting and ploughing, mitigating surface degradation and stabilizing frictional behavior. Furthermore, higher impact resistance reduced microcrack propagation and delamination, enhancing tribological durability. In this case, random jute composite with 30 wt.% (31.8 kJ/mm^2^) provides more strength than a pure random kenaf composite with 23.3 kJ/mm^2^, while a particulate hybrid random composite with 27.5 kJ/mm^2^. It has been proposed that additional fillers can be used to increase the tribological behavior and moisture resistance, for example, SiC, carbon, and glass fillers, tamarind kernel powder hybrid natural fiber composites^92,93^. Table 9 presents summary of earlier studies regarding the impact of natural fibers and fillers on the tribological performance of polymer composites.

**Table 9:** Summary of earlier studies regarding the impact of natural fibers and fillers on the mechanical and tribological performance of polymer composites.

Material	Tensile strength (MPa)	Flexural strength (MPa)	Impact strength	Water absorption (%)	Hardness	Tribology performance SWR/COF	Ref
Snake grass + Luffa-SiC	59.22 ± 3.9	78.63 ± 0.7	2.3 J ± 0.060	7.5	–	–	^94^
Glass + Jute + Kenaf-SiC hybrid composite	87	123	–	–	71	–	^95^
Sisal + Basalt Powder	112.2	163.2	4	–	85	125 microns	^96^
Cotton polyester	–	–	–	–	–	3.5 × 10^–5^ mm^3^/Nm/ < 1	^97^
Sisal polyester	–	–	–	–	–	1 × 10^–5^ mm^3^/Nm/ 0.65	^98^
Coir Polyester	–	–	–	–	–	1.7 × 10^–5^ mm^3^/Nm/ 0.66	^99^
Chopped glass fiber polyester						2.85 × 10^–5^ mm^3^/Nm/ 0.68	^100^
Bamboo epoxy	–	–	–	–	–	5 × 10^–5^ mm^3^/Nm/ 0.6	^101^
Cotton 5%Gr + polyester	–	–	–	–	–	1.2 × 10^–5^ mm^3^/Nm/ 0.62	^97^
3%Gr + Date plam epoxy	61	–	–	–	84.88	1.35 × 10^–5^ mm^3^/Nm/ 0.47	^102^
Jute + Kenaf	37	45	50 kJ/m^2^	–	45	2 × 10^–4^ mm^3^/Nm /0.37	^103^
Pineapple + Kenaf	44.13	43.05	58.9 kJ/m^2^	–		9.7 × 10^–5^ mm^3^/Nm/0.213	^104^
Pineapple + Flax + peanut oilcake filler	27.1	56.27	73.23 J/m	6.6	–	3.57 × × 10^–5^ mm^3^/Nm/0.178	^105^
Kenaf + Jute + 1%SiC	141.25	181.5	27.5 kJ/ m^2^	8.4	61.2	3.33 × × 10^–5^ mm^3^/Nm/0.154	Present study

## Conclusion

The use of gray-fuzzy multi-response modeling and optimization is proven to be powerful approach in analyzing the tribological properties of hybrid composites. This method provides a flexible, systematic, and efficient way to optimize for multiple responses. The optimal combination of parameters for fabricating hybrid composites with superior tribological characteristics are determined to be 1 wt% SiC, 30 wt% reinforcement, 1500 m sliding distance, 1.5 m/s sliding speed, and 15 N load for safer operating conditions.

- The results of the ANOVA analysis indicated that the wt% of SiC*SiC had the utmost significance with F value of 22.21, indicating dominance influence on wear performance. SiC is the most sensitive factor among the several influencing factor due to its synergistic effect between fiber and SiC. This synergy facilitates mechanism such as fine debris formation, the ploughing effect, and the transformation of elastic to plastic.
- Nevertheless, increasing SiC nanofiller content beyond optimal level led to particle agglomeration, promoting severe wear attributed to the formation of rough tribo flim under aggressive sliding condition.
- Residual plot provides evidence that the experimental trial data points’ defuzzified output variable was in close proximity to the line of reference. Subsequently, optimal parameter set derived from the grey-fuzzy technique was further evaluated for moisture absorption and post-exposure mechanical properties. Water absorption test indicated that fiber–filler hybridization significantly enhanced moisture resistance by reducing 7.5% water diffusivity and interfacial degradation. Likewise, combination of 1 wt% SiC and 30 wt% fiber reinforcement offered the best trade-off between wear resistance and mechanical strength. These findings underscore that appropriate tmf and precise tuning of fuzzifiers significantly enhance model’s accuracy and effectiveness in optimized tribo-mechanical performance of kenaf/Jute-SiC Hybrid composites
- SEM images of worn-out specimens disclosed the different types of wear mechanisms responsible for the wear-out of specimens.
- The obtained kenaf/jute-SiC filled composite can serve as in the design and development of mild load conditions, which are more representative of light-duty applications such as bearing, appliance sliders, cages, sealing interface where moderate wear resistance and low friction are critical.

## Data Availability

The data that support the findings of this study are available from the corresponding author upon reasonable request.
